# Particle Size Effect of Curcumin Nanocrystals on Transdermal and Transfollicular Penetration by Hyaluronic Acid-Dissolving Microneedle Delivery

**DOI:** 10.3390/ph15020206

**Published:** 2022-02-08

**Authors:** Hong Xiang, Sai Xu, Jingyuan Li, Shihui Pan, Xiaoqing Miao

**Affiliations:** 1 Marine College, Shandong University, Weihai 264209, China; 202017672@mail.sdu.edu.cn (H.X.); 201900810213@mail.sdu.edu.cn (S.X.); panshihui@sdu.edu.cn (S.P.); 2 SDU-ANU Joint Science College, Shandong University, Weihai 264209, China; 201900700199@mail.sdu.edu.cn

**Keywords:** particle size effect, nanocrystals, transfollicular, penetration, polysaccharide microneedles

## Abstract

Microneedles are one promising penetration enhancement vehicle to overcome the stratum corneum skin barrier, which hampers the penetration of drug nanocrystals by transdermal delivery. In order to clarify the particle size effect of nanocrystals on transdermal delivery, 60 nm, 120 nm, and 480 nm curcumin nanocrystals were fabricated and incorporated into dissolving hyaluronic acid polysaccharide microneedles. The microneedles showed good mechanical strength with 1.4 N/needle, possessing the ability to insert into the skin. The passive permeation results showed that the smaller particle size of 60 nm curcumin nanocrystals diffused faster and deeper than the larger 120 nm and 480 nm curcumin nanocrystals with size-dependent diffusion behaviors. Thereafter, higher concentration gradients and overlap diffusional coronas also formed in the skin layers by the smaller-particle-size nanocrystals. Furthermore, the diffusion rate of the smaller particle size of curcumin nanocrystals to the hair follicle was also higher than that of the larger curcumin nanocrystals. In conclusion, the particle sizes of curcumin nanocrystals influenced the transdermal and transfollicular penetration in deeper skin layers

## 1. Introduction

Skin is mainly composed by the stratum corneum (SC), epidermis, dermis, and hypodermis layers. Transdermal delivery offers an attractive route for local and systemic drug delivery. Due to the skin barrier, the skin penetration of poorly soluble drugs has been hindered [1]. Only small molecules (<500 Da) and moderately lipophilic molecules (log P 1–3) are allowed to infiltrate the SC layer [2]. Several strategies have been applied to enhance skin permeability, such as a chemical method penetration enhancer and physical methods including sonophoresis, iontophoresis, and electroporation. However, safety concerns including sterility, burns, immunity issues, and allergic issues have occurred for topical drug delivery.

In recent years, nanocarrier-based topical formulations such as nanoemulsion [3], lipid nanoparticles (NPs) [4], polymeric NPs [5], and solid lipid NPs [4] were used for the transdermal delivery of insoluble drugs due to their advantages of convenience, painlessness, hair follicle targeting, and so on. However, the large-scale use of carrier materials often brings a series of problems such as insufficient drug loading and potential safety concerns. Compared with nano-carrier-based formulations, drug nanocrystals (NCs) feature a small amount of surfactant or polymers and a high theoretical drug loading capacity (almost 100%). In addition, NCs also provide several advantages to overcome the SC barrier, such as the creation of a higher concentration gradient, a target potential to the hair follicle, and the formation of diffusional coronas [6]. The skin penetration and hair follicle accumulation of drug NCs to overcome the SC barrier are affected by concentration [7], particle size [8], carriers [9], excipients [10], etc. Briefly, higher concentration gradients, good skin moisturizing properties, and appropriate excipients enhance the diffusion and distribution of NCs in the skin layers or hair follicles. However, it has been difficult for the integral NCs to penetrate the SC [11].

Microneedles (MNs) are minimally invasive devices that painlessly penetrate the SC and generate micropores in the skin, therefore remarkably enhancing drug delivery. NCs loaded into MNs provide a novel and facile platform for poorly water-soluble drugs [12]. First, MNs effectively deliver hydrophobic drugs in an NC formulation, which greatly widens the scope of MN transdermal drug delivery. Secondly, NCs increase the drug loading of MNs. Thirdly, MNs overcome the SC barrier and deliver NCs directly into the skin, subsequently forming multi-nanodrug reservoirs. Several drugs have been fabricated as NCs and loaded into MNs, including curcumin, albendazole, rilpivirine, and cholecalciferol. The MN delivery system can significantly improve the delivery efficacy of drugs loaded with the NCs formulation [13]. The drug release from MNs is a complex multi-factor process. First, aqueous microchannels form after the microneedles insert into the skin, and then NCs are released from the dissolving MNs and drug-rich reservoirs are generated. Driven by the concentration gradient formed by NC reservoirs, the drug NCs gradually dissolve and continuously diffuse in the hydrophilic deep skin layers. This process is affected by the microenvironment of the aqueous microchannel dimensions, characteristics of the polymer of the MNs, and characteristics of the NCs, such as physical and chemical characteristics of the drug molecule, particle size, surface charge, and stabilizers [14]. However, studies on the permeation and HF targeting mechanisms of NCs have mainly focused on topical formulations such as gels and suspensions. A study by Pelikh demonstrated that the skin penetration route of CUR-NCs was strongly influenced by excipients and moisturizing carriers, which may be used to achieve targeted skin drug delivery [9,10]. However, the penetration of CUR-NCs was mainly hindered by the SC. The MNs combined with CUR-NCs can overcome the barrier of the SC and effectively deliver NCs. However, the penetration, distribution, retention, and HF accumulative behaviors of NCs delivered by MNs are not clear. In this article, we combined CUR-NCs with MNs to explore the effect of MNs on the skin distribution of CUR-NCs. Understanding the fate of NCs delivered by MNs in deeper skin layers is of great significance for understanding the behaviors of NCs released from the dissolving MNs.

Therefore, the aim of this study was to explore the size factor influence on the skin penetration, diffusion, and retention mechanism in deeper skin layers. The CUR-NCs with 60 nm, 120 nm, and 480 nm were fabricated and loaded into hyaluronic acid (HA) MNs by topical drug delivery, to clarify the penetration and diffusion corona caused by the concentration gradient in the skin layers formed by different NC particle sizes, and the relationship between particle size and hair follicle accumulation.

## 2. Results and Discussion

### 2.1. Characterization of CUR-NCs

**Figure 1:**
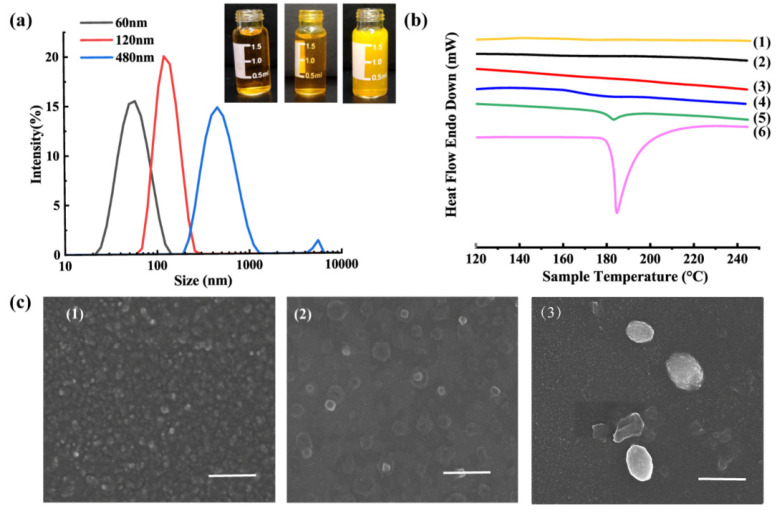
(**a**) The size distribution and photographs of three CUR-NC suspensions; (**b**) DSC profile of PVP (1), 60 nm CUR-NCs (2), 120 nm CUR-NCs (3), 480 nm CUR-NCs (4), physical mixtures (5), and raw CUR (6); (**c**) SEM morphologies of 60 nm CUR-NCs (1), 120 nm CUR-NCs (2), and 480 nm CUR-NCs (3) (scale: 500 nm).

Three particle sizes of CUR-NCs were successfully prepared by the anti-solvent precipitation method (Figure 1a). The particle sizes of CUR-NCs were 60 ± 1.7 nm, 116 ± 7.3 nm, and 488 ± 8.5 nm, while the PDIs were 0.141 ± 0.031, 0.075 ± 0.023, and 0.223 ± 0.005, respectively. The DSC profiles of raw CUR, CUR-NCs, PVP, and physical mixture are shown in Figure 1b. Raw CUR displayed an endothermal peak at 183 °C, while PVP did not display a typical endothermal peak. The physical mixture exhibited a characteristic endothermal peak at 183 °C, as with the CUR raw powder. The endothermal peaks of different CUR-NC particle sizes disappeared, which indicated that the amorphous state was formed. CUR-NCs had a spherical morphology photographed by SEM (Figure 1c), and the sizes of NCs were comparable to the particle size measurement results.

### 2.2. Morphology and Structure of the MNs

**Figure 2:**
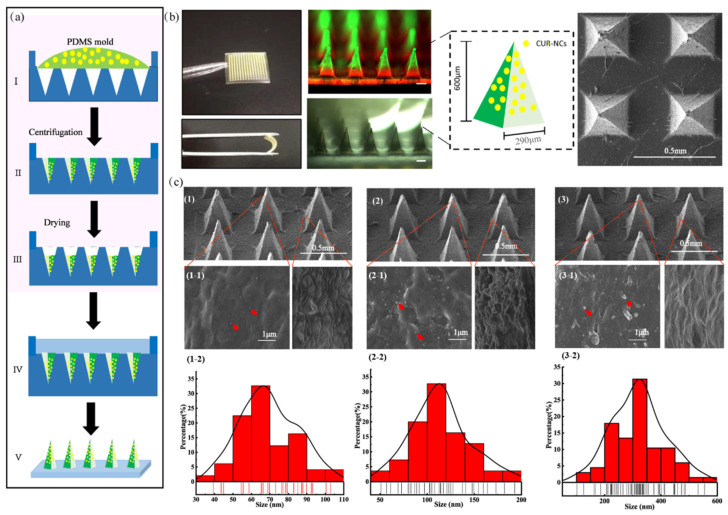
(**a**) Preparation of MNs; (**b**) fluorescent and stereomicroscope image of the MNs (scale: 200 μm); (**c**) SEM images of MNs (1, 2, and 3); magnified SEM images of MNs-tip and MNs-side (1-1, 2-1, and 3-1; red arrows: CUR-NCs embedded in MN-tips) and size analysis results of CUR-NCs embedded in MN arrays (1-2, 2-2, and 3-2).

The three-particle-size CUR-NCs were loaded into the HA-MNs according to the two-layer casting method shown in Figure 2a. The bright and fluorescence-field images of CUR-MNs are shown in Figure 2b. The MNs comprised 225 needles in a 15 × 15 pattern. Each needle had a pyramidal morphology with a height of 600 μm and a base width of 290 μm.

The microstructure of MNs was observed by SEM. As shown in Figure 2c(1) (2),(3), the tip shape of three MNs was pyramidal. The magnified SEM images of the tips and sides of the three MNs are shown in Figure 2c(1-1),(2-1),(3-1). Different sizes of CUR-NCs can be observed in the magnified image at the MNs-tips (red arrows: CUR-NCs embedded into the MNs-tips). The size of CUR-NCs embedded in the MN-tips was then analyzed by Nano Measure software. The analysis results showed that the particle size of CUR-NCs embedded into the MNs-tips was 64.5 ± 7.2 nm (Figure 2c(1-2)), 113 ± 5.3 nm (Figure 2c(2-2)), and 317 ± 22.3 nm (Figure 2c(3-2)), respectively. This result was comparable to the previous particle size distribution results, which indicated that CUR-NCs were successfully incorporated into MNs.

### 2.3. Mechanical and Insertion Properties of CUR-MNs

**Figure 3:**
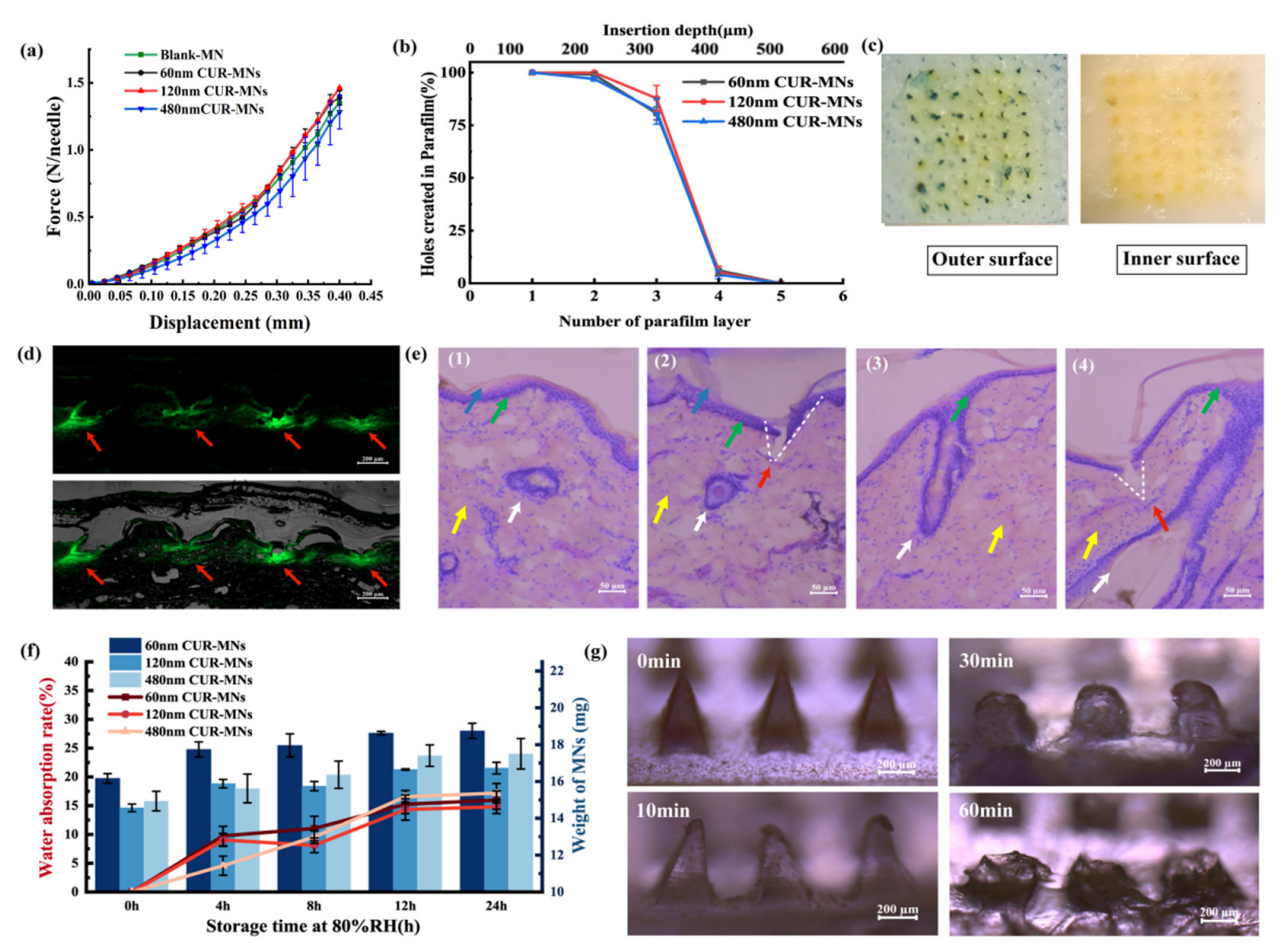
(**a**) Force–displacement curve of MNs. (**b**) The percentage of holes created in Parafilm layers following insertion of MNs (left axis), the number layers of parafilm penetrated by the MNs (bottom axis), and the insert depth of MNs in parafilm (upper axis). (**c**) Outer and inner surface of porcine after MNs insertion. (**d**) Fluorescence and brightfield merged images of vertical skin sections (40 μm) after MNs application. (**e**) H&E staining of skin and hair follicle (blue arrow: SC; green arrow: epidermis; yellow arrow: dermis; white arrow: hair follicle; red arrow: the caves pierced by MNs). (**f**) Hygroscopic analysis of MNs at 80% RH atmosphere, the water absorption rate of MNs (left axis, red, dot-line graph), and the weight of MNs (right axis, blue, histogram) as a function of time (n = 3). (**g**) The solubility of MNs in the skin.

The force–displacement curves are shown in Figure 3a. The mechanical properties of blank-MNs were not significantly different from those of CUR-MNs, indicating that the properties of MNs were not affected by CUR-NCs. Three CUR-MNs tolerated compressive forces of ≥0.14 N needle^−1^, which is expected to enable piercing of the skin [15]. In addition to the mechanical strength, the insertion ability of CUR-MNs was further evaluated. As with the mechanical characteristics assessment, all CUR-MNs were able to insert in three layers of Parafilm M, as shown in Figure 3b. The height of the single-layer Parafilm film was 126 μm, and the penetration of needles to the third layer indicated that the MN inserted up to 378 μm of the total 600 μm height. MNs were also successfully inserted into the porcine skin, and the micropores that formed on the skin surface (Figure 3c) confirmed that MN has sufficient insertion properties (the skin insert rate is 96.9 ± 1.2%).

The images of skin histological slices following insertion of MNs are shown in Figure 3d,e. The H&E results showed that porcine skin was mainly composed by the SC (~22 μm, blue arrow), epidermis (~23 μm, green arrow), dermis (~750 μm, yellow arrow), and HF (the white arrow). MNs can insert into the SC successfully and implant into the dermis (Figure 3e, the red arrow), confirming that the tips of MNs were well penetrated into the skin.

### 2.4. Hygroscopicity and Solubility of MNs

The hygroscopicity of the MNs was evaluated by exposing MNs to high-humidity conditions. The water absorption of the MNs patch was about 15% (Figure 3f) after 24 h, which may be caused by the high hygroscopicity of HA. In addition, the solubility of the MNs in the skin and PBS was studied, and the results are shown in Figure 3g and Supplementary Materials Video S1. MNs completely dissolved in the skin after 1 h. Compared with the slow dissolution in the skin, the MNs dissolved in PBS within 1 min.

### 2.5. Ex-Vivo Transdermal Permeation

**Figure 4:**
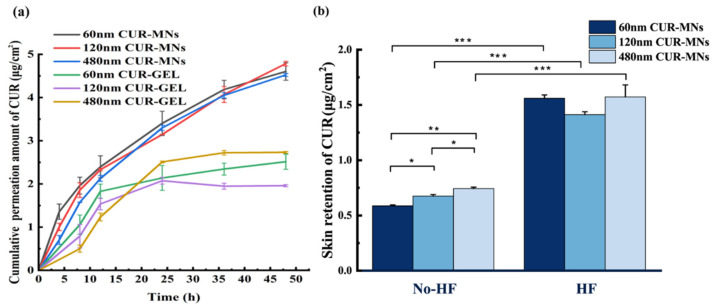
(**a**) Cumulative transdermal permeation of CUR from the MNs in 20% PEG solution (n = 3), and (**b**) the CUR retention in the HFs and skin without HFs. (* *p* < 0.05, ** *p* < 0.01, *** *p* < 0.001 (n = 3)).

The CUR permeated amount of CUR-NCs gel (CUR-GEL) and CUR-MNs after 48 h is shown in Figure 4a. The cumulative CUR released from CUR-MNs after piercing the porcine skin was about two-fold higher than that of CUR-GEL after 48 h. In addition, the small-particle-size CUR-MNs (60 nm) showed a faster and deeper increase in CUR penetration in the first 4 h, where the permeated amount was more than 1.5- and 2-fold higher than those of the 120 nm and 480 nm ones. Compared with the 120 nm and 480 nm CUR-NCs, 60 nm NCs possessed a higher concentration gradient (△C, the concentration difference of CUR between the reservoir area and the subcutaneous area) in the early time due to the higher apparent solubility (Cs) [16]. This concentration gradient contributes to higher passive diffusion through the skin according to Fick’s first law (Equation (1)). However, with the further diffusion of CUR in the skin, the △C between two regions gradually decreased, and the passive diffusion advantage of 60 nm CUR-NCs gradually decreased at 12 h. In this period, the 120 nm and 480 nm CUR-NCs with lower Sc made increasing diffusion behaviors due to the drug release from the reservoir.

(1) $$J=K_{P}\times △C$$

*J*: the flux of drug through the skin; *K_P_*: permeability coefficient, Δ*C*: concentration gradient of drug in skin.

To compare the retention behaviors of different-particle-size CUR-NCs in the HFs and skin without HFs (no-HF), the skin was separated by a scalpel following the permeation of 48 h. The retention rates (retention rate = the total CUR retention/diffusion area) of different-particle-size CUR-NCs are shown in Figure 4b. The retention amount of the three-particle-size CUR-NCs in HF was about 2.3-fold higher than that of the skin (on-HF), which indicated that a large amount of CUR-NCs accumulated in the HF by dissolving HA-MNs by transdermal delivery. In addition, the skin (no-HF) retention of CUR for the 60 nm CUR-MNs, 120 nm CUR-MNs, and 480 nm CUR-MNs was 0.60 ± 0.008, 0.69 ± 0.013, and 0.75 ± 0.013 μg/cm^2^, respectively. Large-sized NCs showed a higher skin retention (*p* < 0.01) compared to smaller-sized NCs. For the HF part, there was no significant difference in the CUR retention of three CUR-MN formulations.

### 2.6. Dermal Penetration of CUR-MNs

**Figure 5:**
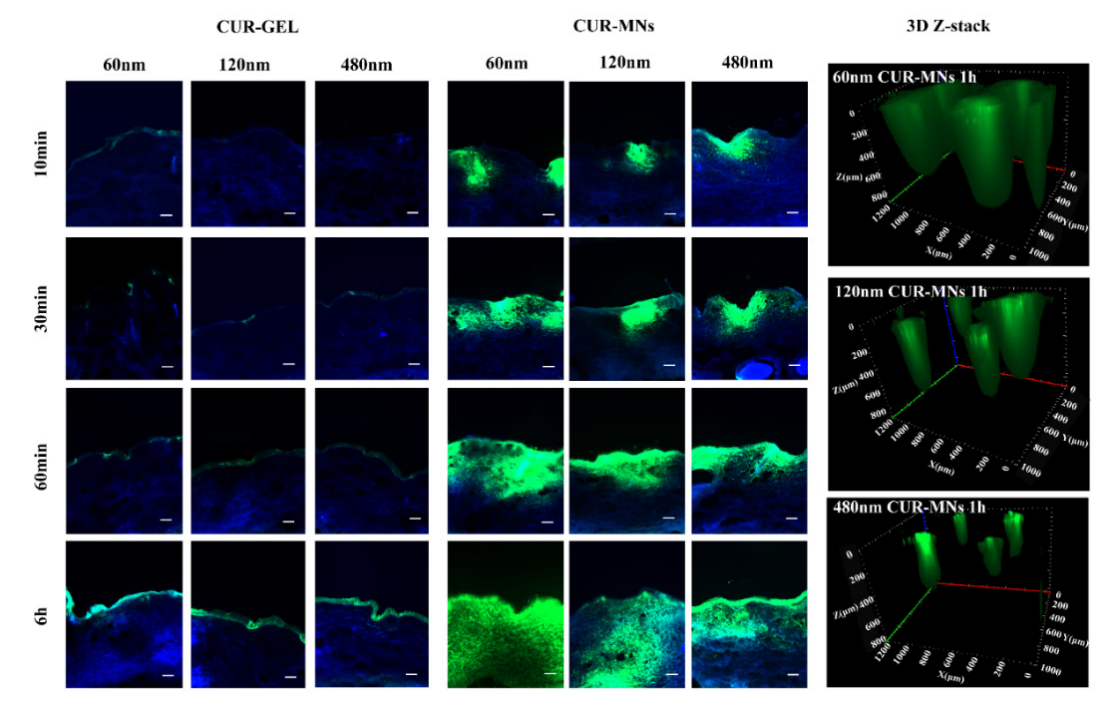
Passive, dermal penetration of CUR-NCs with three particle sizes from MNs and gel (scale bar: 100 μm). Three-dimensional (3D Z-stack) images of three CUR-MNs at 1 h after skin penetration.

The merged and 3D z-stack images are shown in Figure 5 (individual images, as shown in Supplementary Materials, Figures S1–S5). For the CUR-GEL group, most of the green fluorescence appeared in the SC layer instead of entering the viable epidermis, indicating that the CUR-NCs showed poor passive diffusion. These results are in agreement with the recent literature on the transdermal penetration of CUR-NCs [9,10,11]. Compared with the gel vehicle, the MNs facilitated the CUR-NCs delivery into the deeper skin layers. The fluorescence of CUR appeared in the epidermis layer in the first 10 min and the entire skin after 6 h. In the skin penetration of the three-dimensional (3D) scan shown in Figure 5, the diffusional coronas formed with different diameters. Notably, the CUR fluorescence was found in all skin layers after using 60 nm CUR-MNs for 1 h, indicating the effective permeation of NCs with smaller size in the skin with a deeper diffusional corona. With the particle size of CUR-NCs increased, the diffusional corona decreased. For the 480 nm NCs, fluorescence was seldom observed in deep skin layers, indicating that the larger size of NCs tend to accumulate in the skin.

**Figure 6:**
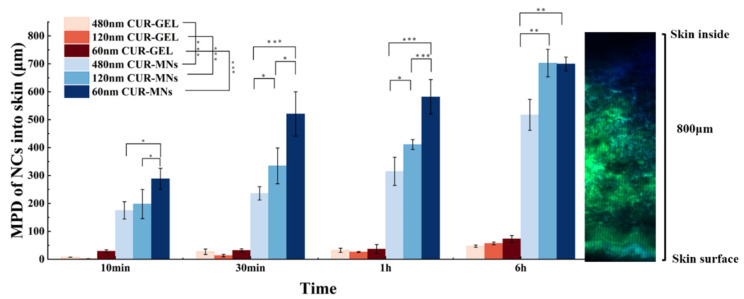
Influence of the particles size on the dermal, passive penetration efficacy (mean penetration depth (MPD)) of CUR from CUR-MNs and CUR-GEL. The image on the right shows the skin penetration image of 60 nm CUR-MNs for 1 h (total skin height is approximately 800 μm). (* *p* < 0.05, ** *p* < 0.01, and *** *p* < 0.001 (n = 3)).

Digital analysis was employed to transfer the observations from visual inspection into objective numbers. As shown in Figure 6, the digital image indicated that the skin passive penetration of NCs remarkably increased by MNs delivery compared with the GEL vehicle. Furthermore, the penetration behavior of three-particle-size CUR-NCs displayed a significant difference. The MPDs of 60 nm, 120 nm, and 480 nm CUR-MNs at 1 h were 581 ± 61.92, 411 ± 17.68, and 315 ± 50.5 μm, respectively, which indicated that CUR-NCs with the smaller particle size were easier to penetrate in the skin layers than the larger particle sizes. Compared with larger NCs, the small particle size of CUR-NCs possessed a higher curvature [16]. According to the Kelvin equation (Equation (2)), the vapor pressure (dissolution pressure) is positively related to curvature. Increasing the dissolution pressure will result in a higher apparent solubility (Sc) [17]. In addition, NCs with a small particle size had a larger specific surface area, permitting the faster dissolution of drugs. Following the insertion of CUR-MNs into the skin, aqueous microchannels were formed and then the HA base was hydrated and fully dissolved; therefore, the CUR-rich reservoirs were generated in the hydrophilic microchannels. Compared with 120 nm and 480 nm CUR-NCs, the higher Sc and specific surface area of 60 nm CUR-NCs led to a higher concentration gradient, which may contribute to a higher passive diffusion in the skin.

(2) $$lnP/P_{0}=\frac{2\gamma V_{m}}{rRT}$$

*P*: actual vapor pressure, *P*_0_: saturated vapor pressure, *γ*: surface tension, *V_m_*: molar volume of the liquid, *R*: gas constant, *r*: radius of the droplet, *T*: temperature.

Another possible reason may be related to the cell uptake and efflux mechanism of NCs under the skin. Generally, NCs with a particle size of less than 100 nm enter a cell through clathrin-mediated endocytosis, non-clathrin-mediated endocytosis, or macro-pinocytosis pathways and then deliver the drug into the underlying tissues. By contrast, NCs with large particle (size > 100 nm) may enter the cell via phagocytosis [18,19]. NCs of small particle size may reach deeper skin through multiple mechanisms of cell uptake and efflux. At present, limited investigations have been reported for the uptake of nanocrystals by cells after topical application. More in vivo experiments on intracellular uptake are needed to elucidate the cellular mechanisms involved in determining the in vivo fate of topically applied nanocrystals.

### 2.7. Hair-Follicle Accumulation of CUR-MNs

**Figure 7:**
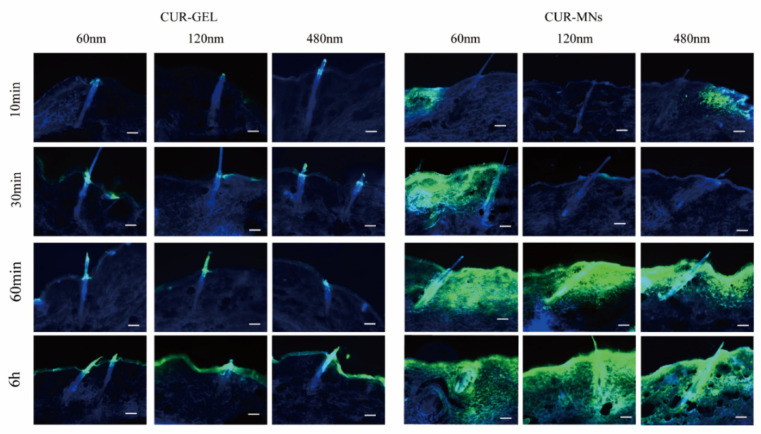
Penetration and accumulation of CUR-NCs into the hair follicles from MNs and gel vehicles (scale bar: 100 μm).

In recent years, HF represented an important penetration pathway for transdermal drug delivery [20]. Unlike the NPs for targeting HF through the follicle opening and penetration along the follicular duct, the CUR-NCs delivered by dissolving MNs showed that transfollicle accumulation depended on the NCs diffusion behavior. The CUR fluorescence of CUR-GEL only appeared in the lower part of the infundibulum. However, the CUR fluorescence of the CUR-MNs group was observed in all areas of HF, including the lower part of the infundibulum, the sebaceous gland, the bulge region, and the hair bulbs, as shown in Figure 7. In addition, compared with 120 nm and 480 nm CUR-NCs, the 60 nm NCs accumulated in the HF in the early 30 min.

**Figure 8:**
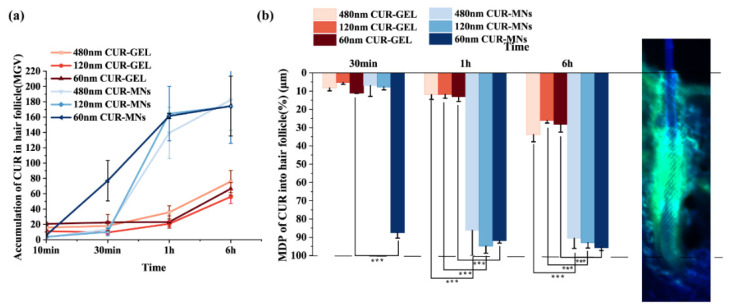
(**a**) Total amount of CUR accumulation released from CUR-MNs and CUR-GEL in HF; (**b**) mean penetration depths (MPDs) of CUR into the hair follicles from CUR-GEL and CUR-MNs. The picture on the right shows the HF and the penetration image of CUR in the HF (*** *p* < 0.001 (n = 3)).

The digital image analysis results of CUR accumulation in HF are shown in Figure 8. The total amounts of CUR accumulation and penetration depth into the HF were estimated by the value of MGV and MPD. For the total accumulative amount of CUR in HF, there were no significant differences in the MGV of three CUR-MNs formulations at 6 h (174.3 ± 78.99 MGV/pixel of 60 nm CUR-MNs, 174.0 ± 78.13 MGV/pixel of 120 nm CUR-MNs, and 182.3 ± 32.88 MGV/pixel of 480 nm CUR-MNs). These results indicated that the accumulative amount of CUR in HF were not affected by the particle sizes. However, in the GEL group, the larger 480 nm CUR-NCs in the gel showed a higher MGV value (76.0 ± 14.36 MGV/pixel), compared to the smaller particle size, including 120 nm gel (56.0 ± 8.69 MGV/pixel) and 60 nm gel (66.5 ± 8.20 MGV/pixel). These results are similar to those of the previous studies that the NPs size between 400 and 700 nm are easy to accumulate in the HF by topical application [21]. However, the accumulative rate in HF of CUR is greatly influenced by the particle size. After 30 min, the MGV value of 60 nm CUR-NCs (77.0 ± 26.29 MGV/pixel) in HF was significantly higher than those of 120 nm (10.15 ± 4.85 MGV/pixel) and 480 nm (13.4 ± 3.98 MGV/pixel), which may be related to the rapid diffusion of small-sized NCs. A study on the delivery of liposome nanoparticles via MNs showed that nanostructured lipid carriers enhance the affinity between the nanoparticle and hair follicles, and enhance hair follicles targeting [22]. One possible reason for CUR accumulation in HF might be the lipid-enriched environment of follicles, which facilitated the accumulation of the lipid-soluble drug.

## 3. Materials and Methods

### 3.1. Chemicals and Materials

CUR (purity > 98%) was purchased from Feiyubio Biological Technology Co, Ltd. (Nantong, China). HA was purchased from Yinhe Biological Technology Co, Ltd. (Shanghai, China). Polyethylene glycol 400 (PEG400) was purchased from China National Pharmaceutical Group Co, Ltd. (Beijing, China). Polyvinylpyrrolidone (PVP) was purchased from Macklin (Shanghai, China). All other chemical reagents were of analytical or chromatographic grade.

### 3.2. Fabrication of Three-Particle-Size CUR-NCs

CUR-NCs with three particle sizes were prepared by the anti-solvent precipitation method, as previously reported [23,24]. Briefly, 10 mg of CUR was dissolved in 2 mL of ethanol as the organic phase and 60 mg of PVP was dissolved in water as the aqueous phase (the volume of aqueous phase was 13 mL for the ~60 nm formulation and 8 mL for the ~120 nm formulation). The organic solution was then injected into the aqueous solution under continuous sonication (JY92-IIDN Huixi, Shanghai, China). The power, sonication time, intervals time, and total sonication time were 400 W, 3 s, 2 s, and 5 min for the ~60 nm formulation; and 400 W, 3 s, 10 s, and 5 min for the ~120 nm formulation, respectively. CUR-NCs with 480 nm were prepared by stirring precipitation. Briefly, 2 mL of CUR (10 mg/mL) was injected into 8 mL of aqueous phase containing 0.12% PVP, under 500 rpm stirring. The organic solvent was removed by 30 kDa Amicon^®^ Ultra-15 centrifugal filter device (Merck, Darmstadt, Germany).

### 3.3. Characterization of CUR-NCs

The particle size and polydispersity index (PDI) of CUR-NCs were measured by a Nano^®^ Zetasizer (Malvern Instruments, Worcestershire, UK). The morphologies of the CUR-NCs were observed by a Nova Nano SEM (FEI, Hillsboro, OR, USA). The thermal properties of the CUR, PVP, physical mixture of CUR and PVP, and three particle sizes of CUR-NCs were measured by a Diamond TG/DTA (Perkin Elmer, Waltham, MA, USA). All the experiments and measurements were performed in triplicate.

### 3.4. Fabrication of CUR-MNs

MNs were prepared by the two-layer centrifugation method (Figure 2a). Briefly, 1 mL of HA gel (20% (*w*/*v*) 5 kDa and 5% (*w*/*v*) 300 kDa) was mixed with 1 mL of CUR-NCs. The mixture was poured into polydimethylsiloxane (PDMS) molds (15 × 15 pyramid, 600 μm height, and radius of 290 μm). The molds were centrifuged at 4000 rpm for 20 min, the extra mixture was removed, and the molds were dried for 1 h at 38 °C. The loading and scraping process was repeated three times. The backing layer HA (10% (*w*/*v*) 300 KDa and 5% (*w*/*v*) 5 kDa) was added to the molds and centrifuged for 20 min at 4000 rpm. Then, the molds were dried for 12 h at 38 °C. The blank MN patches were fabricated by the same process without CUR-NCs added.

### 3.5. Morphology and Structure of CUR-MNs

The morphology and structure of CUR-MNs were observed by an Axio Observer inverted fluorescence microscope (Carl Zeiss AG, Oberkochen, Germany) and Nova Nano SEM (FEI, Hillsboro, OR, USA).

### 3.6. Mechanical Properties Assessment of CUR-MNs

The mechanical force of MNs was tested by a TMS-pilot texture analyzer (FTC, VA, USA), as previously reported [25]. Displacement and force tests began when the sensor first touched the MNs tips and continued until the sensor moved 0.4 mm from the MNs tips toward the backing layer.

### 3.7. Insertion Properties of CUR-MNs and H&E Staining

MNs were inserted to the porcine skin and Parafilm^®^ M using a thumb [26,27]. Bama miniature pig skin (about one-month-old; the hair was carefully removed by a spatula) was purchased from Jingde Agricultural Products Sales Co., Ltd. (Hebei, China), and frozen and stored at −20 °C until further use. The Parafilm^®^ M was unfolded and the number of holes in each layer was calculated under a stereoscopic microscope. The insertion efficiency was calculated by dividing the holes of Parafilm^®^ M in each layer.

The skin specimens were embedded with tissue freezing medium (O.C.T. Compound Leica, Mainz, German) and cut into 8 and 40 μm slices. The 8 μm sections were subjected to H&E staining and the 40 μm section was subjected to skin insertion visualization, respectively.

### 3.8. Detection of Drug Loading in MNs

The needle and the backing layer were dissolved in 20% (*v*/*v*) PEG aqueous solution. The CUR drug loading in the MNs was calculated by subtracting the CUR in the backing layer from the CUR in MNs.

### 3.9. Hygroscopy of MNs

The hygroscopic analysis of MNs was conducted under 80% relative humidity (RH) at 25 °C for 24 h (saturated ammonium sulfate solution). The water absorption of MNs was measured by the weight of MNs at predetermined times. The mechanics of MNs was detected by a Texture Analyser after 24 h.

### 3.10. ExVivo Transdermal Permeation

The transdermal permeation of CUR-MNs was investigated by a vertical Franz diffusion cell with a diffusion area of 2.25 cm^2^. The skin was washed by normal saline and fixed between the donor and the receptor chambers. In this study, 5% propylene glycol with 25 μg of CUR-NCs was set as a control. PEG 400 with 20% (*v*/*v*) was used as a receptor medium to ensure sink conditions. The experiment was carried out at 100 rpm at 37 ± 1 °C (to obtain skin surface temperature of 32 °C). At a predetermined time, 1 mL of receptor medium was withdrawn from the receptor chambers and fresh medium was immediately added, and the collected samples were centrifuged at 12000 rpm for 20 min and analyzed by High-Performance Liquid Chromatography (HPLC).

In addition, after 48 h of permeation, the hair follicle part (HF biopsy) and nonhair follicle part (non-HF biopsy) were separated from the skin by a scalpel. The skin samples (HF biopsy and non-HF) were homogenized with methanol and centrifuged at 12,000 rpm for 20 min. All samples are then tested by HPLC. Measurements were performed in triplicate.

### 3.11. Ex Vivo Model for Passive Dermal and Transfollicular Penetration

The passive diffusion and transfollicular penetration of CUR-MNs were tested by the ex vivo porcine skin model. Briefly, skin was pre-equilibrated in normal saline (32 °C) for 10 min. CUR-MNs were inserted into the skin using a thumb, and then the samples were incubated at 32 °C [9]. At 10 min, 30 min, 1 h, and 6 h, the skin was cleaned with swabs and immediately embedded in tissue freezing medium. The frozen skin was cut into 40 μm slices by a microtome (Leica CM1950, Mainz, Germany).

The passive diffusion and the transfollicular penetration of CUR was detected by an inverted fluorescence microscope (Carl Zeiss AG, Oberkochen, Germany). The skin samples incubated for 1 h were performed in three-dimensional (3D) scanning, and the 3D x-y-z image was constructed from Z-stack images The gamma of the AF488 channel was set to 1.2 to quench the autofluorescence of the skin. In order to visualize the hair follicles in the skin, the DAPI channel was used to excite the autofluorescence of follicles.

### 3.12. Digital Image Analysis

The mean penetration depth (MPD) and mean gray value (MGV was used to estimate the total penetration of CUR in skin) of NCs in the hair follicles and dermis were measured via the software Image J according to a previous study [7]. All the experiments and measurements were performed in triplicate.

### 3.13. HPLC Analysis

CUR was detected by an HPLC system (Agilent 1100, Santa Clara, CA, USA) at 425 nm. Separation was performed on a XB-C18 column (Ultimate^®^; 150 × 4.6 mm, 5 μm) with methanol and 3.6% (*v*/*v*) glacial acetic acid solution (75/25, *v*/*v*) as the mobile phase, and the flow rate was set as 1 mL/min.

### 3.14. Statistical Analysis

All data are presented as the mean ± standard deviation (SD). The results were evaluated by one-way ANOVA. Significance is denoted in the figures as * p < 0.05, ** p < 0.01, and *** p < 0.001.

## 4. Conclusions

In this study, the CUR-NCs (60 nm, 120 nm, and 480 nm) were fabricated and embedded into the tip of MNs. Compared with traditional transdermal delivery, MNs avoid the barrier of the SC. In vitro permeation and retention studies showed that CUR-MNs enhanced CUR permeation, especially through the HF pathway. Compared with the larger size of CUR-MNs, the 60 nm CUR-NCs displayed a faster permeation in the skin layers and HF accumulation. However, the accumulative amount of CUR in HF was not affected by the particle sizes after 6 h. Those may be related to the regulation of skin diffusion caused by the size effect. The small size of NCs formed a higher concentration gradient and overlapped diffusional coronas in the skin, which further enhanced the distribution and diffusion of drugs. The delivery of effective doses of the drug to the HF is very important to treat HF-related diseases, such as acne, androgenetic alopecia, and other sebaceous gland dysfunctions. However, CUR-NCs delivered by gel mainly accumulated in the upper infundibulum of the HF, rather than the deep follicular papilla. The MNs combined with NCs provided a new strategy to treat HF disease.

Therefore, the MNs loaded with NCs can tremendously influence the dermal penetration efficacy and HF accumulation of CUR-NCs. In addition, the particle size will also influence the penetration, accumulation, and retention behavior of CUR-NCs in the skin layers and may thereafter affect the efficacy of drugs. By controlling the proper particle size of drug NCs and matrix materials of MNs, the promising expectations of drug control release may be obtained to treat skin topical and systemic diseases.

## Data Availability

Data is contained within the article and supplementary material.

## Supplementary Materials

The following supporting information can be downloaded at: www.mdpi.com/article/10.3390/ph15020206/s1, Figure S1: Untreated skin and hair follicles; Figure S2: Passive skin penetration of CUR-NCs gel (DAPI and AF488 channels); Figure S3: Cumulative amount in HF of CUR-NCs gel (DAPI and AF488 channels); Figure S4: Passive skin penetration of CUR-NCs MNs (DAPI and AF488 channels); Figure S5: Cumulative amount in HF of CUR-NCs MNs (DAPI and AF488 channels); Figure S6: The effect of HA concentration and molecular weight on the mechanical strength of MNs; Video S1: The solubility of the MNs in PBS.
