# Supplementary material for: Particle Size Effect of Curcumin Nanocrystals on Transdermal and Transfollicular Penetration by Hyaluronic Acid-Dissolving Microneedle Delivery
Source: Pharmaceuticals (Basel). 2022 Feb 8;15(2):206. doi: 10.3390/ph15020206 (PMC8878115; doi:10.3390/ph15020206)
Supplement: Supplementary file 1 [file pharmaceuticals-15-00206-s001.zip › pharmaceuticals-1560822-supplementary.pdf]

pharmaceuticals

Article

Particle Size Effect of Curcumin Nanocrystals on Transdermal and Transfollicular Penetration by Hyaluronic Acid-Dissolving Microneedle Delivery

Hong Xiang [1], Sai Xu [1], Jingyuan Li [2], Shihui Pan [1] and Xiaoqing Miao [1,*]

[1] Marine College, Shandong University, Weihai 264209, China; 202017672@mail.sdu.edu.cn (H.X.); 201900810213@mail.sdu.edu.cn (S.X.); panshihui@sdu.edu.cn (S.P.)
[2] SDU-ANU Joint Science College, Shandong University, Weihai 264209, China; 201900700199@mail.sdu.edu.cn
* Correspondence: xiaoqingmiao@sdu.edu.cn

Supplementary Material

Figures S1–S6

Overview

Figure S1. Untreated hair follicles.

Figure S2. Passive skin penetration of CUR-NCs gel (DAPI and AF488 channels).

Figure S3. Cumulative amount in HF of CUR-NCs gel (DAPI and AF488 channels).

Figure S4. Passive skin penetration of CUR-NCs MNs (DAPI and AF488 channels).

Figure S5. Cumulative amount in HF of CUR-NCs MNs (DAPI and AF488 channels).

Figure S6. The effect of HA concentration and molecular weight on the mechanical strength of MNs.

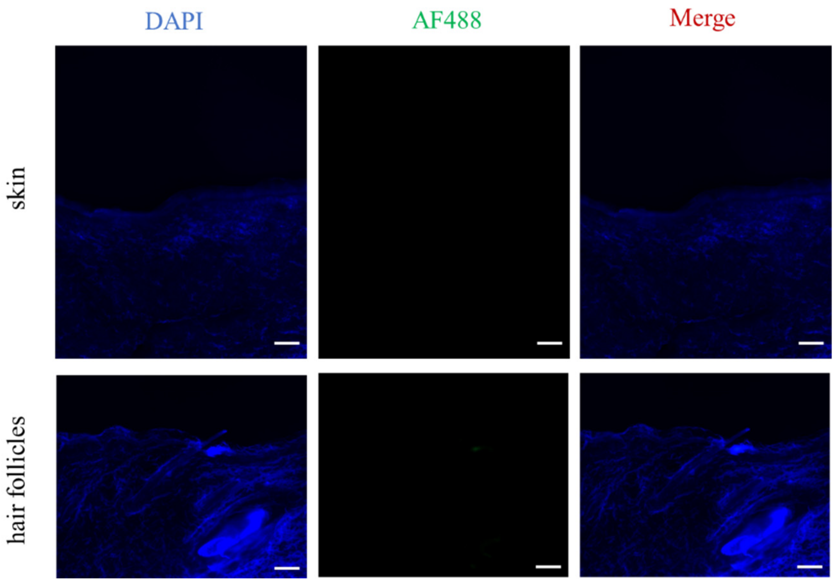

Figure S1. Untreated hair and hair follicles. (Scale bar:100μm).

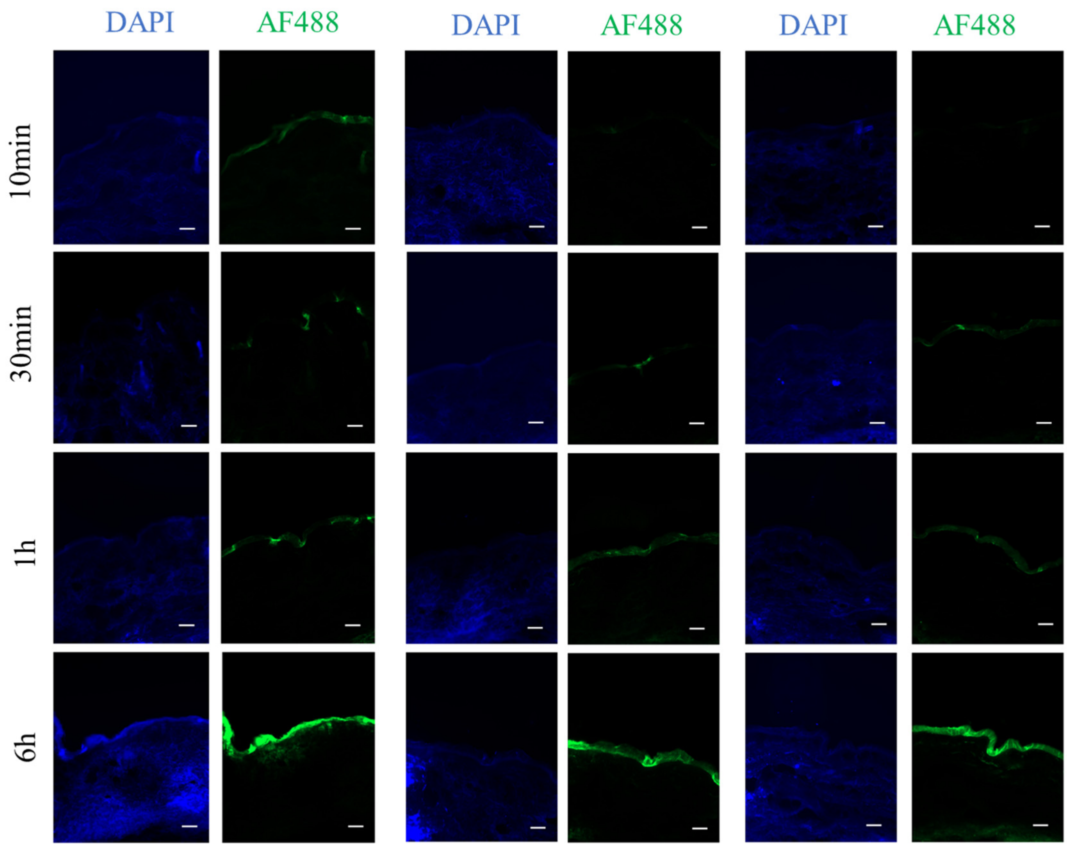

Figure S2. Passive skin penetration of CUR-NCs gel (DAPI and AF488 channels).

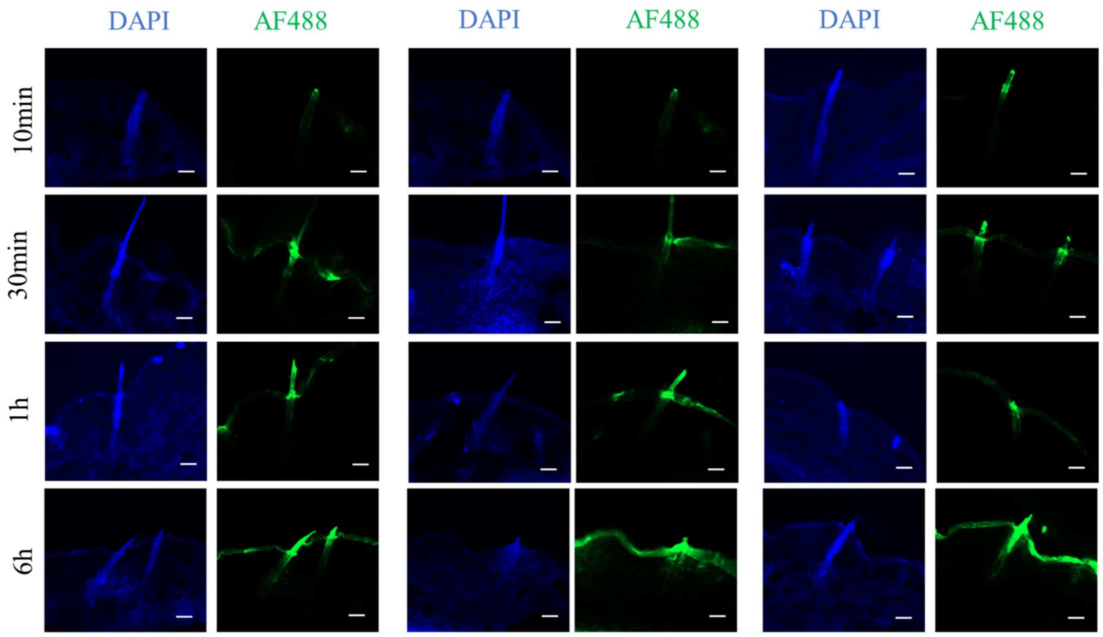

Figure S3. Cumulative amount in HF of CUR-NCs gel (DAPI and AF488 channels).

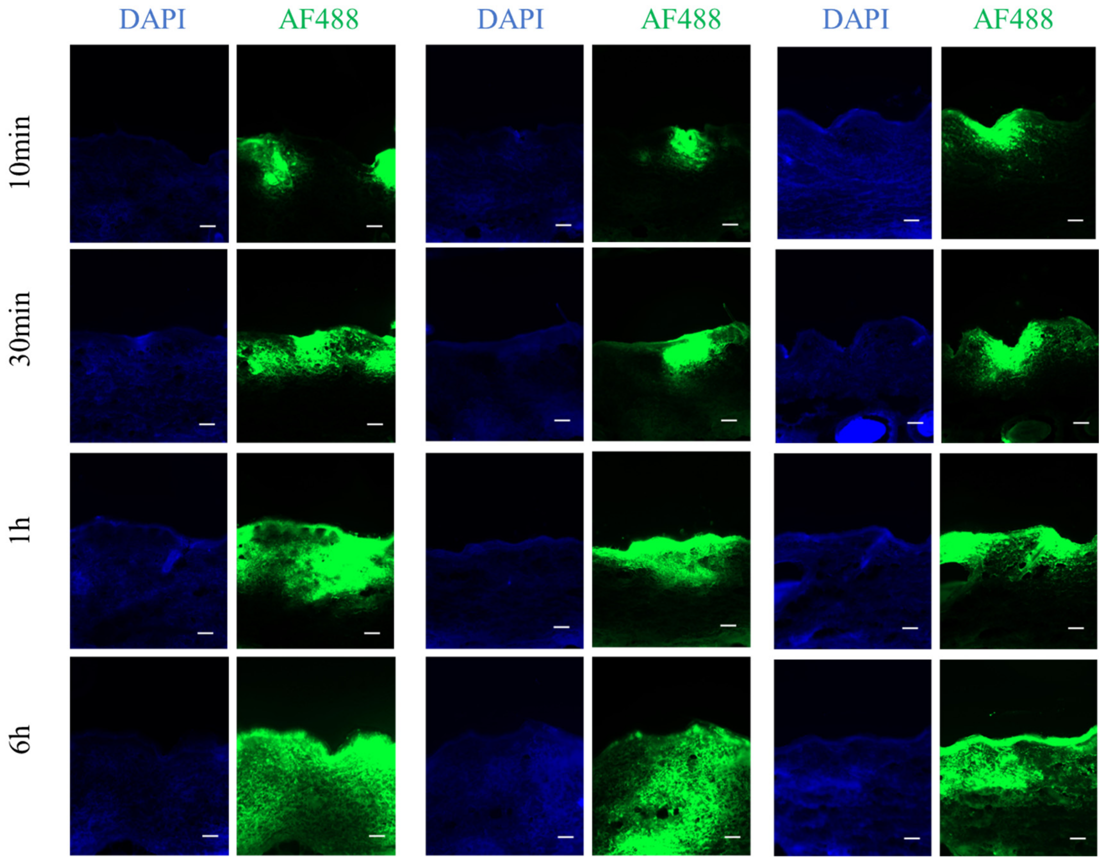

Figure S4. Passive skin penetration of CUR-NCs MNs (DAPI and AF488 channels).

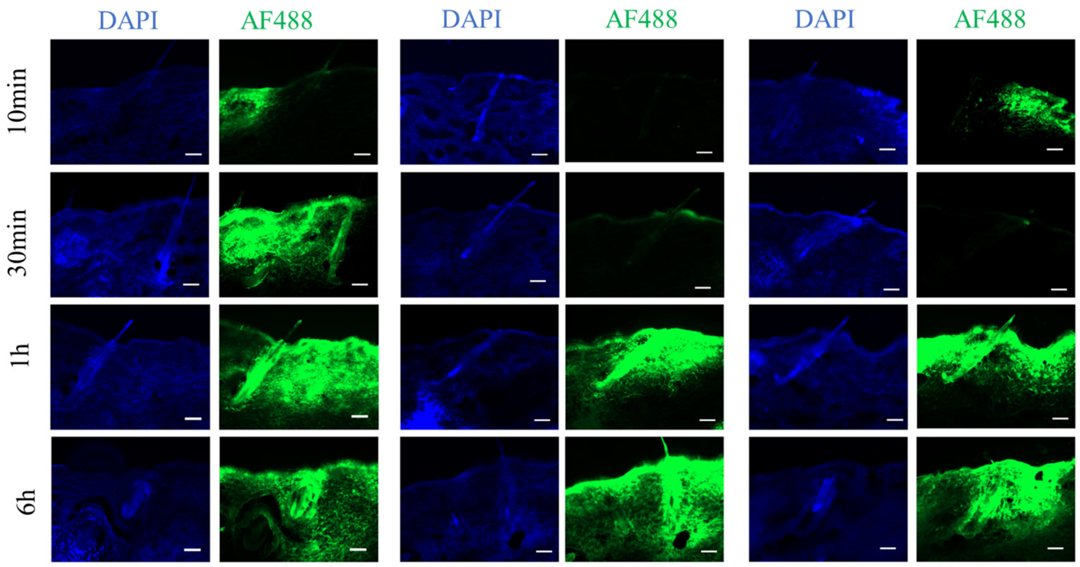

Figure S5. Cumulative amount in HF of CUR-NCs MNs (DAPI and AF488 channels).

Selection of MNs Materials

In our previous study, the effects of HA concentration and molecular weight on MNs properties were investigated.

Firstly, we select one proper HA concentration with good mechanical properties. The force-displacement of different concentration with 15%, 25% and 50% of HA for the same molecular weight of 5KDa were evaluated with by TMS-pilot texture analyzer (Figure S6). These results showed that the mechanical strength of the MNs was gradually increased with the increase of HA concentration. The hardness of 50% and 25% HA-MNs were significantly higher than 15% HA-MNs. In addition, the hardness of 50% HA-MNs was slightly higher than that of 25% HA-MNs, with no significant difference. Considering the mechanical strength and drug loading issues, we choose the concentration of HA with 25% as the matrix of the MNs.

Secondly, the force-displacement the different molecular weights of 5KDa, 300KDa, and 1500KDa with 25% HA concentration were evaluated, respectively. However, 25% 300KDa HA and 25% 1500KDa HA were too viscous to form a homogeneous solution. Therefore, the solution of 25% 5KDa HA, 20% 5KDa HA+5% 300KDa HA and 20% 5KDa HA+2.5% 300KDa HA+2.5% 1500KDa HA were prepared to study the effect of molecular weight of HA on MNs. The force-displacement curves are shown in Figure S6, the results showed that the hardness of the MNs increased firstly and then decreased with the increasement of the molecular weight, and the formula of 20% 5KDa HA+5% 300KDa HA displayed better hardness. Therefore, we select 20% 5KDa HA+5% 300KDa HA as the matrix of HA MNs.

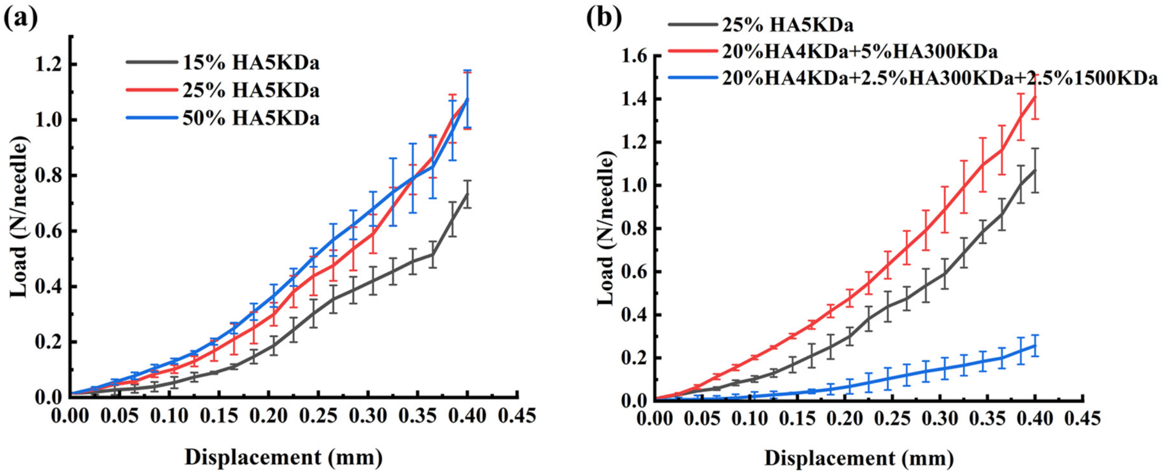

Figure S6. (a) The effect of HA concentration on the mechanical strength of MNs; (b) Influence of HA molecular weight on the mechanical strength of MNs.
